# An Update on Common Pharmaceuticals in the Prevention of Pancreatic Cancer

**DOI:** 10.7759/cureus.25496

**Published:** 2022-05-30

**Authors:** Candace Miyaki, Launa M Lynch

**Affiliations:** 1 Internal Medicine, Idaho College of Osteopathic Medicine, Meridian, USA; 2 Pharmacology, Idaho College of Osteopathic Medicine, Meridian, USA

**Keywords:** pancreatic cancer prevention, metformin, aspirin, carcinoma, pancreas

## Abstract

In this review, we aim to update readers about the most recent studies on common pharmaceuticals and their association with pancreatic cancer risk. The use of prophylactic aspirin, metformin, beta-blockers, and statins has been studied in the past but showed inconclusive results in the reduction of pancreatic cancer incidence. However, in recent studies, these medications along with combination therapy of aspirin and metformin were found to have a more significant association with decreasing risk. Given the poor prognosis of pancreatic cancer despite treatment, medication prophylaxis prevention should be considered. In this review, we hope to encourage future case-control or prospective studies on common medications that have shown great potential in delaying pancreatic cancer development.

## Introduction and background

Pancreatic adenocarcinoma is one of the most lethal cancers in the United States with a five-year survival rate of 10.8% [1,2]. Due to its delayed diagnosis, aggressive nature, and common recurrence, remission is less feasible. In 2021, new cases rose to over 60,000 and deaths over 48,000; with its increasing incidence, poor early detection, and fatal prognosis, focus on prevention of cancer development has been studied [1,3,4]. There are well-known modifiable risk factors associated with increased cancer incidence including smoking, obesity, and alcohol consumption [5-8]. Recent studies are now focusing on pharmaceutical effects on delaying carcinoma onset or overall prevention.

A previous review in 2016 presented data and proposed mechanisms of common pharmaceuticals such as pancreatic carcinoma prophylaxis including aspirin (ASA), metformin, beta-blockers, and statins [9]. Current research has shown more associations between pharmaceutical prophylaxis and cancer risk reduction--the most promising being the combination of ASA and metformin [10-13]. Throughout this paper, we will expound on data initially presented by Amin et al. and update the readers on newer studies done on these pharmaceuticals, both more recent and that which were not previously mentioned. Included is the mechanism of action of each pharmaceutical drug in the setting of pancreatic cancer prevention.

## Review

Aspirin

ASA has shown protective effects against colorectal, gastric, and esophageal cancers [14-16]. Its success with other carcinomas has provoked studies to observe its protective effects against pancreatic cancer [17]. A 2016 review done by Amin et al. indicated conflicting results in terms of ASA use and reducing pancreatic cancer risk [9]. At the time, studies did not show a significant association between the two but did suggest high doses of ASA had the potential of reducing pancreatic cancer incidence. Recent studies and analyses have shown statistically significant evidence that ASA may lead to a reduction in pancreatic cancer incidence.

ASA’s role in the mechanism of pancreatic cancer prevention has not been established; however, theoretically, it regulates the hallmarks of cancer by suppressing tumor cell proliferation and metabolism, decreasing tumor angiogenesis, blocking platelet activation leading to cancer proliferation, and targeting oncogenes in signaling pathways that activate carcinoma progression [18,19].

In 2019, a systematic review of observational studies done by Sun et al. showed ASA usage may lower the risk of developing pancreatic cancer. They found that both high and low frequency ASA use could significantly decrease pancreatic cancer risk (OR = 0.67, 95% CI = 0.51-0.87, P = 0.003 and OR = 0.76, 95% CI = 0.62-0.95, P = 0.015, respectively). The study also conveyed that the duration of five or more years of ASA use was associated with a decrease in pancreatic cancer incidence (OR = 0.76, 95% CI = 0.64-0.91, P = 0.003) [20]. However, a study by Cook et al. showed that low-dose ASA at 75-100 mg daily over a duration of 10 years did not decrease pancreatic cancer incidence. Therefore, there may be a therapeutic window where low-dose ASA proves most beneficial [21].

A meta-analysis done in 2020 by Wu and colleagues suggested that a duration of 10 or more years of >300 mg daily of ASA use may reduce pancreatic cancer risk (OR = 0.73, 95% CI = 0.51-1.04). They also saw that high-dose ASA use was associated with a significant decrease in pancreatic cancer incidence, while low-dose ASA also showed mild benefit compared to those who did not take ASA. However, their consideration of the frequency of ASA use did not show statistical significance. Although results showed a significant association between ASA and pancreatic cancer risk, confounding variables such as smoking, diabetes, and obesity could not be analyzed [22]. ASA treatment in diabetes patients can significantly decrease inflammation, which is a known risk factor for pancreatic cancer [5]. This discrepancy could skew results. Nonetheless, within the studies, there was no evidence of continuous ASA use increasing pancreatic cancer incidence. Due to the severity of the disease and poor prognosis, prophylactic ASA treatment should be considered, and further studies accounting for variables such as smoking, diabetes, and obesity should be considered. From recent studies, it may be worth treating patients with low-dose ASA for 5-10 years, then increasing the dose to >300 mg after 10 years [23,24].

Metformin

Metformin therapy has been associated with decreased risk of breast, liver, and colorectal cancers in patients [25,26]. Potential mechanisms of metformin in the reduction of pancreatic cancer risk have been proposed; in vitro studies suggest proliferation, growth, migration, and invasion of pancreatic cells are inhibited by metformin [27-29]. An alternative mechanism proposed was metformin-induced liver kinase B1 (LKB1) and adenosine monophosphate-activated protein kinase (AMPK) activation, which ultimately led to decreased mammalian target of rapamycin (mTOR), a signaling pathway in carcinogenesis [30,31].

A study done in genetically modified mice models for pancreatic cancer by Chen et al. found that metformin significantly slowed the progression of chronic pancreatitis to pancreatic adenocarcinoma, delayed both early and late pancreatic tumor lesions, and promoted tumor regression, leading to increased survival [31]. In a meta-analysis by Wang et al., data showed a 37% risk reduction of pancreatic cancer with the use of metformin when compared to other diabetic treatments (RR = 0.63, 95% CI = 0.46-0.8, P = 0.003) [32]. Although this study indicated the potential of metformin as a single therapy in the risk reduction of pancreatic cancer, it may be difficult to directly translate results to human models.

A meta-analysis by Li and colleagues showed metformin had significant survival benefits in patients with early-stage pancreatic cancer. In later disease stages, results were more unclear if metformin proved beneficial. It was proposed that due to concentrations of metformin in tumor cells being decreased in late-stage disease, it made it difficult to obtain therapeutic levels in cells. This theory was supported by patients with tumor resection in late-stage disease. With resection, metformin could act directly on remaining circulating tumor cells. Patients in this subset showed increased survival rates; however, survival benefits in this group could also stem from slowing the rate of the aggressive carcinoma by significantly decreasing the macroscopic lesion(s). Li et al. concluded metformin conferred a survival benefit in a dose-dependent manner in early-stage pancreatic cancer. Conversely, two randomized control trials analyzed in the meta-analysis by Li and colleagues showed little to no benefit from metformin with regards to survival, but in these studies, stages were not well controlled. It is possible a significant number of patients were in the metastatic stage. Data from these studies are consistent with the very late-stage disease when compared to cohort studies concluding that metformin provides little benefit during late-stage disease. Due to variable stages and lack of consistent data, it is not plausible to infer that metformin has no survival benefit or prevention effect [33].

Metformin is well tolerated and has shown some benefit in the risk reduction of general cancer incidence [25-33]. Although results have varied in the past, prophylactic metformin treatment in the prevention of pancreatic cancer should be considered. In the interim, further studies including a randomized control trial controlling tumor stage and dosage of metformin should be done [33].

Beta-blockers

Beta-blockers have shown modest potential in risk reduction of colon, breast, and prostate cancers [34]. A plausible mechanism of beta-blocker therapy in the reduction of pancreatic cancer risk is that by inhibiting beta receptors, it could lead to decreased cyclic adenosine monophosphate (cAMP) signaling, and downregulate cAMP-dependent epidermal growth factor (EGF) and vascular endothelial growth factor (VEGF) production [35,36]. This theory showed potential in a study done by Al-Wadei et al., which showed decreased EGF and VEGF levels with propranolol treatments in animals with pancreatic carcinoma [37].

Literature prior to 2015 showed no consensus if beta-blocker therapy had any benefit to the prevention of pancreatic carcinoma. However, in recent studies, Saad and colleagues performed a case-control study in which there was no statistical significance of any prescription of beta-blocker therapy with reduced pancreatic cancer incidence in the short term (OR = 1.06, 95% CI = 0.97-1.16, P = 0.16). The subgroup analysis based on the beta-blocker receptor target indicated considerable reduction with use longer than two years of a non-selective beta-blocker use (OR = 0.75, 95% CI = 0.57-1.00, P = 0.05) [38].

Due to limited data, no obvious consensus can be made on the association between beta-blocker therapy and pancreatic cancer prevention. Nonetheless, recent research has shown some benefits, which warrant further investigation of the duration and frequency of non-selective beta-blocker therapy in the setting of pancreatic cancer risk reduction [38]. There are studies using beta-blockers to treat pancreatic cancer patients that show improved survival [39,40], which is outside of the scope of this article.

Statins

Statin use for the prevention of pancreatic cancer has been debated, as epidemiologic studies published in the literature have demonstrated conflicting results [41-47]. Two cohort studies done in 2018 showed no significant risk reduction [48]. In 2012, a meta-analysis showed no reduction in pancreatic cancer risk with concurrent statin use [49]. However, a recent meta-analysis done in 2018 showed a significant risk reduction of 30% in pancreatic cancer development (OR = 0.70, 95% CI = 0.60-0.97, p < 0.0001) [50]. Recent observational studies have also found statins to have significant benefits in incident risk reduction [51].

The proposed mechanism is the inhibition of 3-hydroxy-3-methylglutaryl coenzyme A reductase, which is necessary for the mevalonate pathway. Inhibiting the mevalonate pathway degrades the mutant tumor suppressor gene, p53, and suppresses the activation of Ras, Rho, and Rac, which play a role in tumor cell survival [51-54].

The most recently reported study done in 2021 by Saito and colleagues found that with one year of statin treatment, the incidence of pancreatic adenocarcinoma was reduced compared to the control group (RR = 0.84, 95% CI = 0.73-0.97). Although this study was done solely with the Japanese population, it did minimize race variables. Heterogeneity may be the cause of the discrepancy between results amongst previously published studies [51].

Another notable control study showed the significant potential of statins in reducing pancreatic cancer development and concluded benefits could be dependent on duration. In patients that prophylactically took statins for six months or longer, there was a 67% risk reduction in carcinoma development. Although this study did control variables such as smoking and diabetes, it did not control dosage or genetic predisposition [55]. These are important factors to consider controlling in future studies, as both can greatly skew results.

Recent studies done in 2019 used statins to treat pancreatic cancer patients, which improved overall survival [56]. However, further discussion was omitted, as the content is outside of the scope of this article.

Aspirin and metformin

When used individually, ASA and metformin proved to have some benefits with pancreatic cancer risk. Recent studies have shown that when combined together, they have a synergistic effect with decreasing carcinoma incidence [13]. The proposed mechanism given by Yue and colleagues is the inhibition of cell migration in PANC-1 and BxPC-3 cells in mice model studies. They also suggested that the combination could significantly decrease myeloid leukemia-1 (Mcl-1) and B-cell lymphoma-2 (Bcl-2), which are important regulators in cancer cell survival [57]. Mice treated with ASA or metformin alone did not show any decrease in Mcl-1 or Bcl-2. This demonstrated a possible synergistic approach to preventing pancreatic cancer [11]. A brief summary of the mechanism of actions of pharmaceuticals in the setting of pancreatic cancer risk reduction is included in Table 1.

**Table 1:** Proposed mechanisms of action of pharmaceuticals in pancreatic cancer prevention. ASA, aspirin; LKB1, liver kinase B1; AMPK, adenosine monophosphate-activated protein kinase; mTOR, mammalian target of rapamycin; cAMP, cyclic adenosine monophosphate; EGF, epidermal growth factor; VEGF, vascular endothelial growth factor; Mcl-1, myeloid leukemia-1; Bcl-2, B-cell lymphoma-2.

Pharmaceutical	Suggested mechanism of action	Reference
ASA	Suppress tumor cell bioactivity, deteriorate tumor microenvironment, block platelet activation, and target oncogenes in signaling pathways	[18,19]
Metformin	LKB1 and AMPK activation leading to decreased mTOR	[30,31]
Beta-blockers	Decreased cAMP signaling and downregulate cAMP-dependent EGF and VEGF production	[35,36]
Statin	Inhibiting 3-hydroxy-3-methylglutaryl coenzyme A reductase leading degradation of mutant p53 and decreased Ras, Rho, and Rac	[51,52]
ASA and metformin	Inhibition of PANC-1 and BxPC-3 and decrease in Mcl-1 and Bcl-2	[11]

A study done in 2015 found that in cells treated with ASA or metformin alone, the transcriptome profile of PANC-1 was minimally changed, while over 1,000 genes were significantly affected when treated with metformin and ASA together. Treatment at low concentrations of combined ASA and metformin decreased the viability of PANC-1 and BxPC-3 mice cells. With the combination, there was also an upregulation of nerve growth factor receptor (NGFR), which acts as a tumor suppressor in the bladder, stomach, liver, colorectal, and prostate. Although it is difficult to translate mice models to human models, this combination has shown benefits in human studies [12].

Sung et al. saw the potential benefit of this combination in patients. Those that received both ASA and metformin had the most significant risk reduction in overall cancer development compared to ASA or metformin alone (HR = 0.53, 95% CI = 0.45-0.63; HR = 0.79, 95% CI = 0.71-0.88; and HR = 0.80, 95% CI = 0.73-0.87, respectively), as shown in Table 2 compared with all other pharmaceuticals discussed in this review. There were some uncontrolled variables in the study, including obesity, diet, alcohol consumption, and smoking. However, this should not discount the potential of using these drugs as preventative measures, especially in patients with increased risk factors such as smoking or genetic predisposition [10].

**Table 2:** Statistical effects of pharmaceuticals on pancreatic cancer risk reduction. OR, odds ratio; RR, relative risk; ASA, aspirin; Meta, meta-analysis; HR, hazard ratio; CC, case-control; Co, cohort study.

Pharmaceutical	Variable	Effect, 95% CI	P-value	Study type	Reference
ASA	High frequency	OR = 0.67	0.003	Meta	[20]
	Low frequency	OR = 0.76	0.015	Meta	[20]
	5+ years	OR = 0.76	0.003	Meta	[20]
	10+ years	OR=0.73	-	Meta	[22]
Metformin	NA	RR = 0.63	0.003	Meta	[32]
Beta-blockers	Short term	OR = 1.06	0.16	CC	[38]
	2+ years	OR = 0.75	0.05	CC	[38]
Statin	NA	RR = 0.84	-	Co	[51]
	NA	OR = 0.70	<0.0001	Meta	[50]
ASA and metformin	ASA alone	HR = 0.79	-	Co	[10]
	Metformin alone	HR = 0.53	-	Co	[10]
	ASA + metformin	HR = 0.80	-	Co	[10]

There is limited research on the combination of ASA and metformin in the prevention of pancreatic adenocarcinoma; however, due to the significant synergistic evidence shown, it may prove beneficial to continue studies in this area. It may hold great potential in decreasing carcinoma incidence if given prophylactically. Clinical trials to assess the combination of metformin and ASA as prophylactic treatment for patients predisposed to pancreatic cancer are warranted [10,11].

## Conclusions

Pancreatic adenocarcinoma is one of the most lethal cancers. With its increasing incidence and consistently poor prognosis, prevention is a viable aim to decrease pancreatic cancer mortality. There are well-known modifiable factors associated with an increased incidence that can be mitigated by the patient through lifestyle changes. In recent studies, pharmaceutical benefits of risk reduction have been explored. There has been increasing evidence that certain medications hold great potential in decreasing carcinoma incidence, including ASA, metformin, beta-blockers, statins, and, particularly, the combination of ASA and metformin. This review aims to update the reader on current research on these medications and their effect on decreasing pancreatic cancer incidence. Given the low risk profile of the listed pharmaceuticals and significant benefits seen, it warrants further research to understand their potential more clearly in pancreatic carcinoma prevention. Throughout the years, there have been conflicting data regarding each drug and its potential to decrease the incidence of pancreatic cancer. This is likely due to uncontrolled variables in clinical studies and reviews, some that were even referenced in this article; however, this should not discount their merit. To decrease conflicting results, further research controlling smoking, alcohol consumption, drug dosage, drug duration, and disease staging should be considered.
